# Water Temperature From Hot Water Outlets in a Major Public Hospital: How Hot is Our Water?

**Published:** 2011-12-12

**Authors:** Dana Hartley, Ashlee McCarthy, John E. Greenwood

**Affiliations:** ^a^Medical School, University of Adelaide, Adelaide, South Australia; ^b^Adult Burn Centre, Royal Adelaide Hospital, Adelaide, South Australia

## Abstract

**Objectives:** To measure the water temperature issuing from a representative sample of patient-accessible outlets around the Royal Adelaide Hospital. To initiate an audit cycle in the event of unacceptably high water temperatures to ensure their reduction. **Methods:** Samples were taken of water issuing from hot taps, showers, and hand hygiene basins in patient areas throughout the hospital, encompassing newly renovated as well as old sections of the hospital. A 45 second “warming” period was followed by hot water collection into a polystyrene cup to 8 cm. The mean of 3 temperatures measured using an infrared thermometer was calculated. **Results:** Several taps and patient showers were demonstrated to supply water at a temperature capable of causing scald injuries. Most problem outlets were found in the oldest section of the hospital, fewer in the wing of “intermediate” age and none in the most recently renovated emergency block. The data were tabulated and summarized before being passed on to RAH Engineering and Building Services, which initiated remedial action. Several thermostatic mixing valves were found to be faulty (and replaced) or poorly set (and re-set). After this adjustment, outlets previously supplying excessively hot water were re-tested and found to be safe, closing the audit loop. **Conclusion:** Maintenance services infrequently conduct scheduled assessment of hot water outlet temperatures and rely on staff member complaints or concerns to focus their attention on problem areas. With recent evidence of the disastrous potential of hot water on a vulnerable population of hospital in-patients, hazard identification and reporting is everybody's responsibility.

Hot water scalds are among the most common presentations to the Adult Burn Centre of the Royal Adelaide Hospital (RAH). Scalds from hot tap water are a common, yet preventable injury, especially in the young, elderly, and disabled people.^1^ Vulnerability is heightened in hospitals and aged care facilities. Recently, in an Australian institution, an unattended patient collapsed in a ward shower when only the hot tap was on and sustained 70% total body surface area scalds. Complicated by underlying health problems, this became a fatal injury. Hospitals and Aged Care Facilities have since fallen under government and media scrutiny leading to the upregulation of systems to closely control the temperature of hot water at patient outlets.

National legislation on hot water delivery (National Plumbing and Drainage Standard AS/NZS 3500.4: 2003) clearly states the following:

“All *new* heated water installations shall, at the outlet of all sanitary fixtures used primarily for personal hygiene purposes, deliver heated water not exceeding; (a) 45°C for early childhood centres, primary and secondary schools and nursing homes or similar facilities for young, aged, sick or people with disabilities; and (b) 50°C in all other buildings.”^2^

Despite the RAH being built more than 150 years ago, it has a multitier hot water delivery system. Regular and varied checks ensure that the water is heated to, and maintained at, an appropriate level (>60°C) to prevent legionella proliferation in the hot water storage tanks,^3^ yet delivery to the user (patient, health care worker) must be at a temperature not higher than 50°C.^4^ The RAH's hot water is produced and maintained by an experienced engineering faculty. The water within the RAH's hot water duct system is controlled between less than 60°C and less than 80°C via 2 separate systems which use different types of heater, calorifiers, and heat exchangers. Both systems use steam to heat water to 70°C. There are mechanisms in place on both types of water heater to prevent the water from being heated in excess of 80°C. The temperature will then decrease to less than 50°C via the attachment of thermostatic mixing valves (TMVs) fitted to every tap and shower fixture within all patient care areas.

The TMVs in use at the RAH are the Horne 15 TMV H1502 model (Fig 1). These have both hot- and cold-water inputs. Hot water enters and is directed into a mixing chamber, where it meets the cold water before being diverted into a sensing chamber to the outlet. A thermostat element with an actuating pin abuts an adjusting screw. If the water in the sensing chamber is too hot, the thermostat causes the actuating pin to protrude which pushes the valve closer to the hot-water input and away from the cold-water input. This reduces the amount of hot water relative to cold water reaching the mixing chamber and lowers the temperature of the water from the outlet. The temperature at which the valve will control the mixed water is “set” by manipulating the adjusting screw. A failsafe mechanism is inbuilt, so that in the event of cold-water supply failure, the hot-water inlet port closes.

There is an obvious balancing act between the prevention of proliferation of microorganisms and safety, when delivering hot water to patients and health care workers at a hospital. Ultimately, water should never be delivered to the user at a temperature greater than 50°C.^4^

The ability to minimize the number of hot water burns relies greatly on the knowledge of the temperature of water in which a serious burn can occur as well as being aware of those with the highest risk of suffering from a hot water burn.^1^ In 1947, Moritz and Henriques^5^ investigated the relationship between water temperature, time of exposure, and depth of burn. They established that the hotter the water temperature the shorter the time needed to create a burn. Water at 60°C can result in a second-degree burn after 3 seconds, and a third-degree burn after 5 seconds in adults, with shorter exposure times resulting in serious burns in children. At 55°C, deep second-degree burns would occur after 20 to 30 seconds, and still at 49°C a 9-minute exposure time would produce second-degree burns.^5^ These figures have been confirmed more recently.^6^ A recent study in New South Wales, Australia, reported a decrease in hot tap water scalds since the introduction of mandatory regulation on hot water, with a maximum of 50°C, on all newly installed sanitary fixtures.^7^ The RAH aims for a maximum emitted water temperature of 45°C, with a failsafe “cut-off” temperature at 50°C.

Concerns have been raised by some staff members, however, that water from some outlets appears hotter than acceptable and redoubled the need for this investigation, which may have important implications for all institutions where patients, staff, and visitors might be exposed to water temperatures hot enough to cause injury.

## OBJECTIVES

To determine whether the RAH has adequate temperature control of hot water delivered from patient-accessible outlets, or whether patients and staff are at risk of scald injuries within this health care setting.

## MATERIALS AND METHODS

### Taps and showers of the RAH

**Figure 2:**
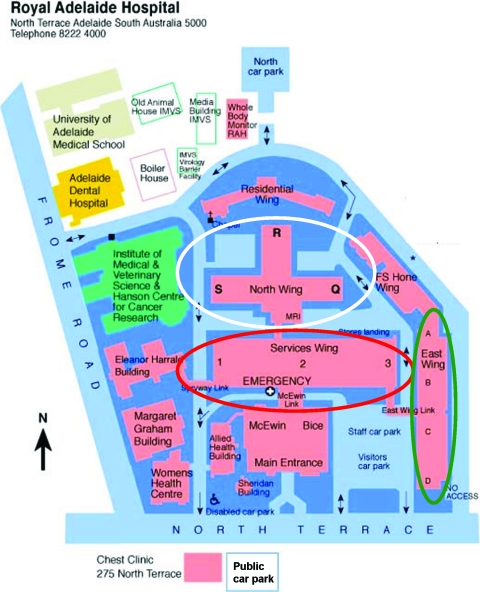
Site map of the Royal Adelaide Hospital indicating the positions of the assessed areas—“old” North Wing in white oval, “intermediate” East Wing in green oval, and newly renovated Emergency Block in red oval.

A measurement of hand basin, shower, and hand station hot water temperatures was obtained from a total of 23 taps, 23 showers, and 49 hand stations taken to be a representative sample reflecting the entire hospital (Fig 2). Seven wards from the North Wing, 5 from the East Wing and the Intensive Care Unit, Emergency Department and Burns Unit from the Emergency Block were involved to ascertain any discrepancy. This was deemed important within the RAH because the different hospital wings not only use different means to heat water but were also built or renovated at different times in the hospital's history.

### Temperature recording

A Testo 830-T1 infrared thermometer with laser sighting was used to measure the temperature of the water. Subjectively, the water approached maximum temperature after 45 seconds of running and this was taken as our sample collection start time. A polystyrene cup (Dart Insulated Foam Cups 177 mL) was filled to a depth of 8 cm with tap or shower water to ensure that a consistent body of water was being measured. The cup was filled and temperature measured 3 times and a mean value calculated. For analysis, the hot water outlets on each ward were separated into categories—ward hand hygiene stations, patient bathroom taps, and patient showers.

## RESULTS

Thirty-nine taps (36%) provided hot water at temperatures in excess of 50°C and 72 (67%) above 40°C (Table 1). Water from 35 taps (33%) was at 40°C or less. Of hot water taps with temperature recordings of more than 50°C, 72% were located in the old North Wing, in which 51% of taps produced water at dangerous temperatures (>60°C). The maximum temperature recorded was 68.5°C. The temperature of water within shower facilities throughout the hospital ranged from 36°C to 65°C, with 83% of shower water temperatures less than 45°C. However, 3 showers (all in the North Wing and 2 on the same ward) recorded temperatures of 55°C, 58.5°C, and 65°C. These temperatures are rapidly capable of creating significant scalds and pose risk to both patient and health care worker. Several problem outlets existed where elderly patient, some with cognitive impairment, were cared for.

Some areas of the RAH demonstrably have insufficient control of water temperature at patient outlets, in particular the older North Wing of the hospital. Twenty-two of 28 ward bay hand stations, used frequently by staff and accessible to patients and visitors, reached temperatures in excess of 63°C.

The newer areas of the hospital with in the Emergency block (renovated in the early 2000s) have effective temperature regulation, with all taps in patient areas measuring less than 41°C. The exception within this area was within the Staff Beverage and Utility rooms (mean 53.5°C), where the water temperatures have been set deliberately higher to allow eating utensil and cup washing, etc. These outlets are not accessible to patients.

## DISCUSSION

A large discrepancy exists between hot water outlet temperatures, which has been demonstrated to be solely due to the physical age of the systems. This is most noticeable in the old North Wing, where even the application of TMVs has not been as effective in controlling temperatures as their application in the more recent hospital buildings. The newly renovated wards are fitted with either automatic sensory, or single piece mixer taps. All the hand hygiene stations within the Emergency Department, Intensive Care Unit, and Burns Unit are fitted with automatic sensory taps which not only effectively control water temperature but the mechanism of action of a sensor “start and stop” function make prolonged contact impossible.

The older wards are fitted with “3-piece” taps, which have a TMV, which can be “set” but must be regularly checked and “re-set” as necessary. Several of the problem taps in the current study had TMVs which were faulty and needed replacement. One of the drivers of this study was the scalding death that occurred in the shower of another institution. The vast majority of the showers assayed at the RAH are satisfactorily controlled, although a small number were discovered to be above a safe temperature level.

The results were presented to the RAH Engineering and Building Services, which immediately launched its own investigation, discovering that some TMVs were faulty and that others were in need of adjustment. In particular, shower facilities were brought under tight control and indeed all problems identified by our investigation received remedial action. In all, 3 TMVs were completely replaced—this takes our service engineers approximately 2 hours to complete and each new TMV costs A$310. All the other problem outlets could be rectified by “resetting” alone (manipulation of the adjusting screw), which takes an experienced engineer approximately 30 minutes for each valve. The remedial action, therefore, was relatively inexpensive once the problem had been identified. However, the engineering and building services admit that they rely on medical/nursing/allied health staff to identify and report problems like overly hot tap water, rather than on repeated, scheduled (or even ad hoc) measurement by their own engineers.

## CONCLUSIONS

In the domestic situation, we take for granted our ability to regulate the temperature of our shower or basin water, protected by our skin's sensory envelope and musculoskeletal escape functions. Those in hospital are vulnerable to many hazards due to age or infirmity and rely upon those caring for them for protection. In this study, problems were identified which, elsewhere, have already claimed the life of one such patient. Similar audits can be performed easily at any health care facility, needing only a thermometer, a protocol for measurement, and the inclination to make the effort to ensure patient safety. It is the role of us all, as carers, to be proactive in safeguarding our charges and in identifying hazards before they cause injury and reporting them to the proper authorities for remediation.

## Figures and Tables

**Figure 1:**
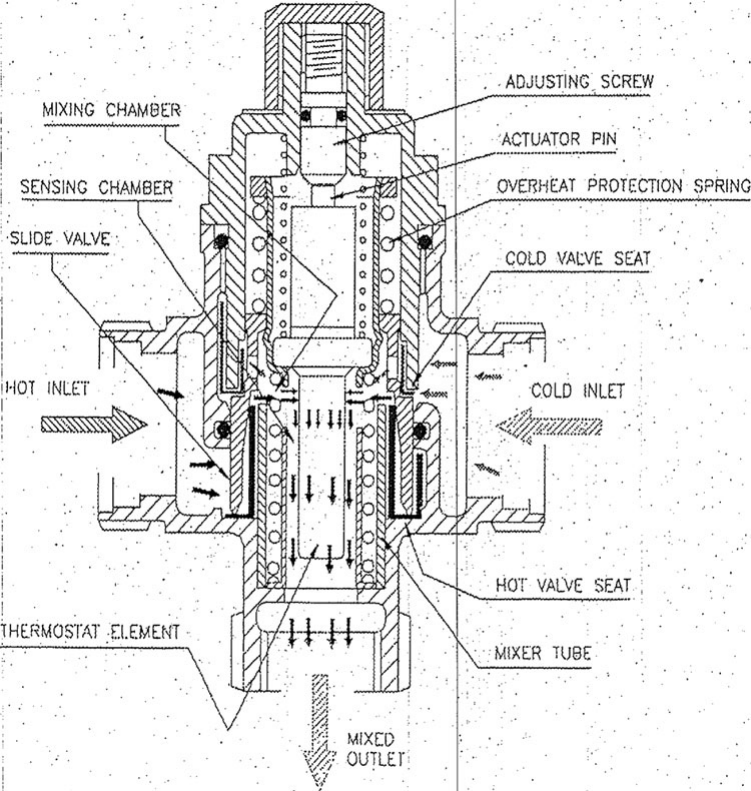
Schematic of the Horne 15 (TMV H1502) TMV in use at the Royal Adelaide Hospital. TMV indicates themostatic mixing valve.

**Table 1:** Recorded water temperatures from “problem” outlets on selected wards and units before and after adjustment

Ward	Tap Location	Survey Temperature, °C	Postadjustment, °C
North Wing	Shower	65	33
	Tap	66	36.5
East Wing	Shower	55	41
	Tap	55.5	41
	Nurses' hand basin	55.5	38.5
	Shower	58.5	38.5
	Tap	60.5	39.0
	Bay 1 tap	55	40.5
	Bay 2 tap	61	38.5
	Bay 3 tap	58.5	38.5
